# Periodontal bacteria influence systemic diseases through the gut microbiota

**DOI:** 10.3389/fcimb.2024.1478362

**Published:** 2024-11-15

**Authors:** Mengying Xi, Qijun Ruan, Sulan Zhong, Jiatong Li, Weijuan Qi, Congman Xie, Xiaoyan Wang, Nuerbiya Abuduxiku, Jia Ni

**Affiliations:** ^1^ Department of Periodontics, Stomatological Hospital, School of Stomatology, Southern Medical University, Guangzhou, China; ^2^ Department of Periodontics, Shenzhen Longgang Otolaryngology hospital, Shenzhen, China; ^3^ Department of Orthodontics, Stomatological Hospital, School of Stomatology, Southern Medical University, Guangzhou, China; ^4^ Department of Stomatology, The First People’s Hospital of Kashi, Kashi, China

**Keywords:** periodontal bacteria, oral dysbiosis, gut dysbiosis, oral-gut axis, systemic diseases, fecal microbiota transplantation

## Abstract

Many systemic diseases, including Alzheimer disease (AD), diabetes mellitus (DM) and cardiovascular disease, are associated with microbiota dysbiosis. The oral and intestinal microbiota are directly connected anatomically, and communicate with each other through the oral-gut microbiome axis to establish and maintain host microbial homeostasis. In addition to directly, periodontal bacteria may also be indirectly involved in the regulation of systemic health and disease through the disturbed gut. This paper provides evidence for the role of periodontal bacteria in systemic diseases via the oral-gut axis and the far-reaching implications of maintaining periodontal health in reducing the risk of many intestinal and parenteral diseases. This may provide insight into the underlying pathogenesis of many systemic diseases and the search for new preventive and therapeutic strategies.

## 1 Introduction

As the largest microbial community in the human body, the gut microbiota plays a crucial role in establishing and maintaining host physiological homeostasis. A variety of human diseases are known to be associated with dysbiosis of the gut microbiota, such as obesity, cardiovascular disease and neurological disorders (Jin et al., 2019; Chen et al., 2021). The proposed theories of gut-liver axis, gut-brain axis, gut-lung axis, and gut-bone axis also fully illustrate the close relationship between the gut microbiota and various organs and systems in the human body (Cryan et al., 2019; Dang and Marsland, 2019; Albillos et al., 2020; Tu et al., 2021). The oral cavity is the second largest microbial habitat in the human body after the gastrointestinal tract (GIT). Studies have shown significant disease-specific patterns in the composition of the salivary microbiota of periodontitis patients (Lundmark et al., 2019). The dominant phyla included Firmicutes, Fusobacteria, Actinobacteria, Synergistes, Spirochetes, Proteobacteria, Saccharibacteria (TM7), and Bacteroidetes are highly associated with periodontal disease, contributing in part to the diversity and functional differences in the oral microbial community (Pei et al., 2020). The oral microbiota can be modified by systemic diseases with increased inflammation, such as diabetes mellitus (DM) and rheumatoid arthritis (RA), which consequently increase bacterial pathogenicity and susceptibility to periodontitis (Graves et al., 2019). Conversely, oral bacteria can impact systemic disease through bacteremia and has been linked to worsening of Alzheimer disease (AD), DM, cardiovascular disease and RA (Hajishengallis et al., 2022; Brewer et al., 2023). More interestingly, oral bacteria may indirectly affect these diseases by influencing the composition of the gut microbiota (Mesa et al., 2019; Sureda et al., 2020; Park et al., 2021; Barutta et al., 2022).

The oral cavity is directly connected to the GIT, and the progression of the ecological niche from the oral cavity to the gut has been defined as the “oral-gut microbiome axis” (hereafter referred to as the “oral-gut axis”). In addition to oral diseases, the oral microbiota is also involved in the regulation of extra-oral diseases through the oral-gut axis. The oral cavity is a microbial reservoir that constantly replenishes the gut microbiota (Schmidt et al., 2019). One study using 16S ribosomal RNA analysis provides evidence of widespread translocation of oral bacteria to the gut. After analyzing 144 pairs of saliva and stool samples, it was found that shared amplicon sequence variants between the salivary and gut microbiota were present in 72.9% of subjects, and that their total relative abundance in the gut was significantly higher in older subjects or those with dental plaque accumulation (Kurushima et al., 2023). Another large-scale controlled study of saliva and fecal samples taken separately from periodontally diseased/healthy subjects confirmed that periodontal status may indeed drive variations in the salivary and gut microbiota (Kurushima et al., 2023). Bao et al. further verified that periodontitis can induce intestinal dysbiosis and inflammation through the influx of salivary microbes by transplanting saliva from patients with periodontitis into mice via oral gavage (Bao et al., 2022). Furthermore, periodontal treatment may improve systemic health through the oral-gut axis further supports this concept and suggests target that periodontal therapy may be systemically useful by reducing the impact of the oral microbiota on intestinal bacteria. Analysis of stool and saliva samples from periodontitis patients using 16S ribosomal RNA gene amplicon sequencing confirmed that periodontal treatment both mitigated oral dysbiosis and altered gut microbial composition (Baima et al., 2024). Other evidence comes from studies examining a positive effect of periodontal therapy on the development of liver disease in cirrhotic patients by reducing bacterial dysbiosis in the feces (Bajaj et al., 2015; Bajaj et al., 2018). The need for a periodontal examination prior to liver transplantation has also been noted (Guggenheimer et al., 2007; Raghava et al., 2013).

How the oral and gut microbiomes interdependently regulate physiological functions to impact systemic health has not been fully investigated. In this paper, we provide potential mechanisms by which periodontal bacteria regulate extra-oral diseases via the oral-gut axis, and the far-reaching implications of maintaining periodontal health in reducing the risk of many intestinal and parenteral diseases.

## 2 Occurrence of periodontal pathogenic bacteria

Host–microbiome homeostasis in the oral cavity can be seen as an ‘armed peace’ that maintains a controlled state of inflammation. The transition from health to disease requires a susceptible host and a dysregulated oral microbiota. For susceptible host, as shown in diabetic mice, diabetes-enhanced IL-17 alters the oral microbiota and renders it more pathogenic (Xiao et al., 2017). On the contrary, local factors, such as poor oral hygiene, may induce oral microbial dysbiosis (e.g. altered proportions of coccobacilli), which ultimately leads to inflammation of the periodontal tissues (Lin et al., 2021). The ensuing increased flow of gingival crevicular fluid not only introduces a component of the host’s defenses, but also provides the substrate necessary for the growth of many ‘inflammophilic’ bacteria (i.e. those that thrive within an inflammatory environment, e.g. *Porphyromonas gingivalis*) (Hajishengallis, 2014). Inflammophilic bacteria actually gain a growth advantage over commensal microbiome in response to inflammation. With the aim of self-feeding, Inflammophilic bacteria develop a range of pathogenic strategies (e.g. affecting neutrophil functions) that gradually disrupt the ecological balance of the existing subgingival microbial community, leading to a more intense host response (Higashi et al., 2024).

The emergence of periodontal bacterial pathogenicity may be related to plaque biofilms attached to the surfaces of teeth, interdental spaces or restorations. Dental plaque represents the microbial community whose collective properties differ significantly from planktonic bacteria, which enhance the survival, metabolism and pathogenesis of oral microorganisms (Freire et al., 2021). Periodontitis-specific pathogenic bacteria, such as *P.gingivalis*, *Treponema denticola, Filifactor alocis, Tannerella forsythia* and *Aggregatibacter actinomycetemcomitans*, colonize and proliferate predominantly within the host periodontal pocket (Darveau et al., 2012). Three key factors may be involved in the induction of inflammation by periodontal pathogenic bacteria to promote periodontal tissue damage, including oral mucosal inflammation and alveolar bone destruction. The first is a change in the relative abundance of dominant species (e.g., inflammophilic bacteria). High levels of the genera *Porphyromonas* (32.2%), *Fretibacterium* (10.4%), *Rothia* (5.3%), and *Filifactor* (3.1%) were observed in periodontitis (Abusleme et al., 2021). Studies have shown that a very small proportion of *Porphyromonas gingivalis* in the community can orchestrate the normal benign microbiota into a dysbiotic community structure (Darveau et al., 2012). The second is related to the location of the plaque biofilm. In line with this view, Carrouel et al. noted that even in periodontally healthy young adults, the interdental space favors the development of periodontal disease as an ecological niche where microbial communities congregate (Carrouel et al., 2016). Compared to supragingival biofilms of other oral mucosa, the interdental biofilm is located between two teeth and the gingiva, where bacteria are in a more anaerobic environment. Due to its unusual anatomy, the body has few or no alternative defenses against the interdental space, and traditional methods of daily control (e.g. toothbrushing and saliva) are inadequate for biofilm disruption at this anatomical location (Carrouel et al., 2016). Quantitative real-time PCR assays have been applied to reflect microbial succession events in developing interdental biofilms, strongly suggesting that oral microbial dysbiosis is associated with the risk of periodontal and related diseases (Carrouel et al., 2016). The final factor is bacterial virulence. For example, gingipains are critical virulence factors for Porphyromonas gingivalis to colonize and proliferate in the gingival crevice and to invade the periodontium (Silva and Cascales, 2021). *F. nucleatum* mediates important biofilm-organizing behaviour and interactions with host cells through the expression of numerous adhesins (e.g. RadD) (Brennan and Garrett, 2019).

Because of the deleterious effects of periodontal pathogenic bacteria, there is increasing interest in whether daily antimicrobial strategies can prevent or reverse oral dysbiosis by altering the structure and function of the plaque biofilm, including healthy diet, oral hygiene, and the use of antimicrobials and probiotics. There have been studies showing changes in the oral microbiota associated with different dietary patterns, such as the amount of fermentable carbohydrates, fats, and anti-inflammatory/pro-inflammatory components, the degree of processing, and supplementation with nitrate (Anderson et al., 2020; Stanisic et al., 2021). An oral health optimized diet (low in carbohydrates, rich in Omega-3 fatty acids, and rich in vitamins C and D, antioxidants and fiber) has been found to reduce the load of potential cariogenic and periodontal bacterial species in the plaque biofilm, and even to reduce gingival and periodontal inflammation in humans (Tennert et al., 2020). However, some human studies have observed no clear relationship between diet and the composition of oral bacterial communities (Santonocito et al., 2022). Scholars holding this view believe that food is actually present in the mouth for a limited period of time and that the primary nutritional sources for oral bacteria appears to be saliva and gingival crevicular fluid. It has also been suggested that some dietary effects on the oral microbiota may occur indirectly by altering the gut microbiota. In conclusion, more research is still needed to elucidate whether healthy dietary patterns can prevent/reverse oral dysbiosis by altering the composition of oral microbiota/plaque biofilms.

In oral hygiene, the use of toothbrushes, interdental brushes, chewing gum, and even anti-plaque agents (e.g. chlorhexidine) are effective in reducing plaque or interrupting plaque maturation, but none of them seem to be able to influence the structure of the bacterial community in plaque. The main reason for this is the blocking effect of biofilms (Rosier et al., 2018). For example, although an anti-plaque agent blocks the formation of plaque, it has little activity against established plaque. The use of probiotics has the potential to prevent and/or treat oral and related diseases by reversing oral dysbiosis. Studies have shown that the addition of probiotics (e.g. *Lactobacillus rhamnosus*) does result in significant changes in the composition of oral bacterial communities, including an increase in microbial diversity and a decrease in the relative abundance of opportunistic pathogens (Di Stefano et al., 2023). It should be noted that this effect may be reversed after a period of cessation of treatment.

## 3 Possible pathogenic mechanisms of periodontal bacteria through the oral-gut axis

**Figure 1:**
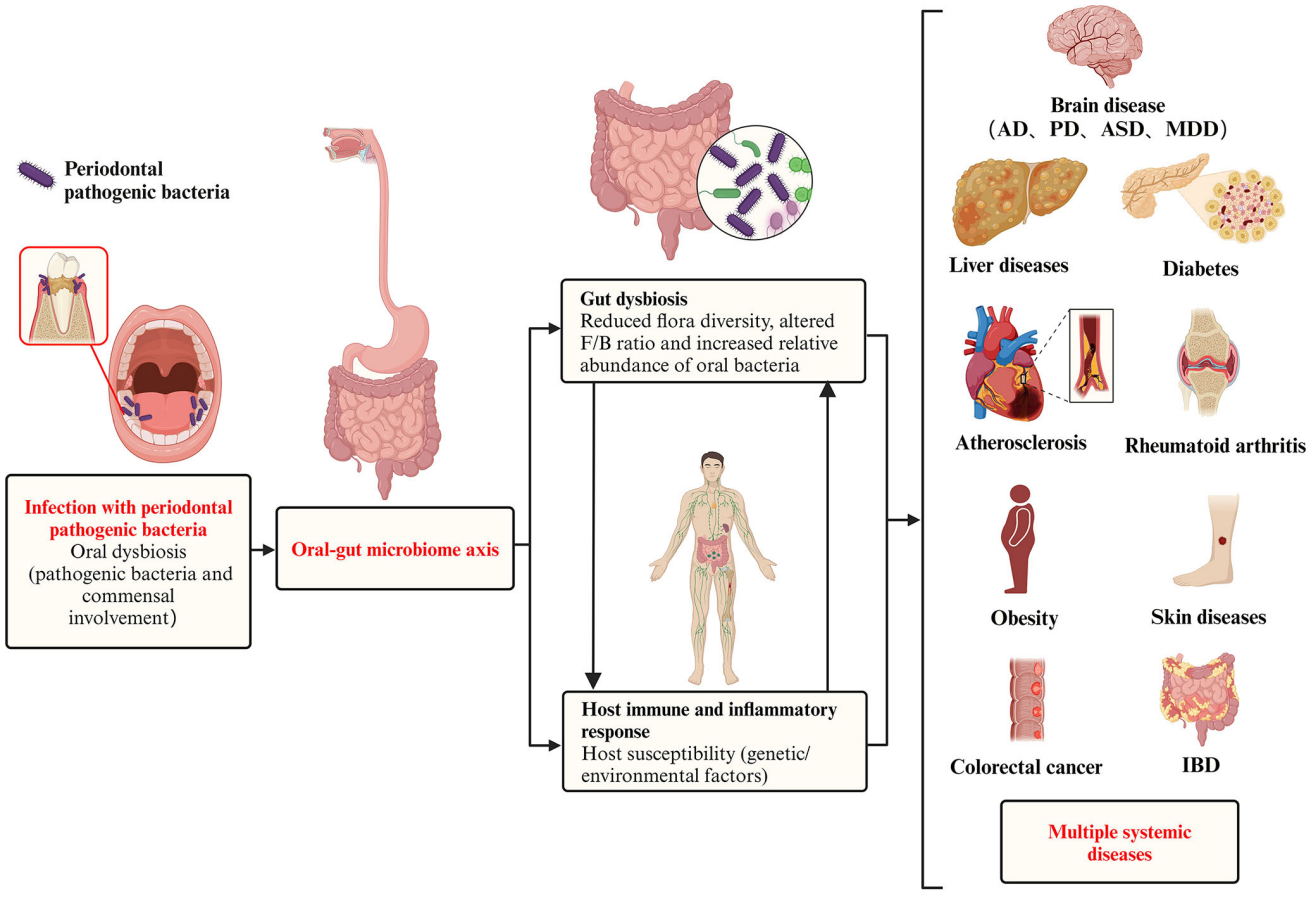
Pathogenic effect of periodontal pathogenic bacteria on systemic diseases via the oral-gut axis. In a pathogenic environment with excessive plaque accumulation, the dynamic equilibrium between microbial invasion and host defense is disrupted, and the periodontal pathogenic bacteria and commensal bacteria together give rise to oral dysbiosis and disease states. The oral cavity is directly connected to the gastrointestinal tract, and the progression of the ecological niche from the oral cavity to the gut has been defined as the ‘oral-gut microbiome axes. These periodontal pathogenic bacteria and their virulence products then breakthrough the barrier between the oral cavity and the gut and translocate in large numbers into the gut, causing gut dysbiosis, which is characterized by (i) a reduction in overall microbial community diversity, (ii) an alteration of the Firmicutes/Bacteroidetes (F/B) ratio, and (iii) as well as a reduction in probiotics and a corresponding increase in opportunistic pathogens. In addition to microbiota dysbiosis, the transition from health to disease requires a susceptible host (including genetic/environmental factors), accompanied by a complex set of interacting mechanisms that ultimately trigger destructive immune and inflammatory responses in the host. In addition to oral diseases, systemic diseases in which the oral microbiota may be involved in regulation via the oral-gut axis include intestinal diseases (e.g. inflammatory bowel disease and colorectal cancer), rheumatoid arthritis, brain diseases (e.g. Alzheimer’s disease, Parkinson’s disease, autism spectrum disorders, and major depressive disorder), liver diseases (e.g. non-alcoholic fatty liver disease, cirrhosis, and hepatocellular carcinoma), obesity, diabetes, atherosclerosis, and skin diseases.

Microbial transmission between the oral cavity and the gut can shape and/or reshape the microbial ecosystem in both habitats, which in turn affects the overall microbial community of the organism. Multiple changes in the gut microbiota, intestinal barrier function and immune system induced by periodontal bacteria can lead to an increased risk of systemic diseases by promoting low-grade inflammation (**Figure 1**). Therefore, exploring the pathogenic role of periodontal bacteria through the oral-gut axis may help us to understand one of several avenues by which periodontitis may affect systemic diseases.

The nature of the dysbiotic change that induces periodontitis has not been well defined and it has been difficult to distinguish between pathogenic and commensal bacteria. However, in the gut, dysbiosis has been identified as a reduction in alpha and beta diversity and an increase in bacterial pathogens, including a decrease in microbial population and functional diversity and stability (e.g. specific alterations in *Peptococcaceae* and *Prevotellaceae*), altered Firmicutes/Bacteroidetes (F/B) ratios, decreased abundance of beneficial SCFA-producing bacteria (e.g. *Blautia*, *Roseburia*, and *Lachnospiraceae*), and a corresponding increase in opportunistic pathogens (e.g. *Megasphaera*, *Enterobacter*, and *Desulfovibrio*) (Sultan et al., 2021; Chidambaram et al., 2022).

The gut originally is colonized by bacteria from the oral cavity or transit through the oral cavity. For example, *Streptococcus salivarius* and *Streptococcus parasanguinis* localized in the oral cavity most often colonize the intestinal niche (Kageyama et al., 2023). In addition, oral bacteria can alter the composition of the gut bacteria either directly or indirectly. In the intestine they may alter commensal bacteria through bacteria-bacteria interactions. For example, potential pathogens entering the gut can signal through quorum sensing to commensals and trigger the expression of toxins, virulence factors, and biofilm formation (Coquant et al., 2021). Periodontal bacteria (e.g. *P. gingivalis*) have been found to express a interspecies quorum sensing signal known as autoinducer-2 (AI-2), which modulates gut microbiota composition by enhancing Firmicutes growth and increasing the F/B ratio (Thompson et al., 2015; du Teil Espina et al., 2019).

**Figure 2:**
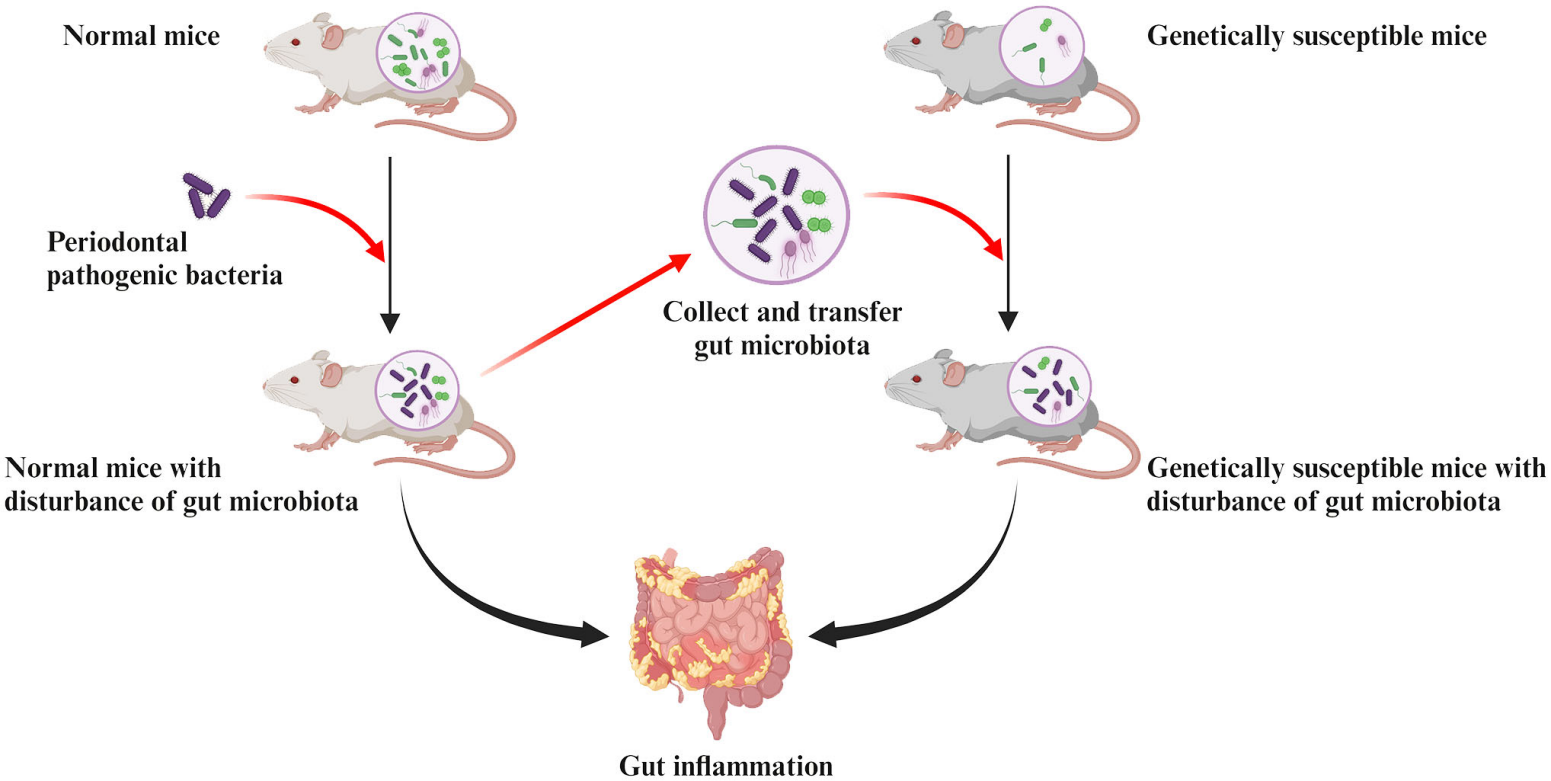
Pathologic mechanisms of intestinal inflammation induced by periodontal pathogenic bacteria are associated with the gut dysbiosis. Collecting the gut microbiota of normal mice after oral administration of periodontal pathogens and transferring it to genetically susceptible mice can observe the same intestinal inflammation in recipient mice. This confirms that the disruption of gut microbiota induced by periodontal pathogens is sufficient to cause their own intestinal inflammation.

A recent mouse study not only demonstrated that oral administration of *P. gingivalis* induced intestinal dysbiosis, reduced intestinal barrier function, and intestinal inflammation, but also further elucidated the pathological mechanisms behind the disruption of intestinal homeostasis by *P. gingivalis* through gut microbiota transplantation (Sohn et al., 2022). This study collected the gut microbiota of normal mice after oral administration of *P. gingivalis* and transferred it to genetically susceptible mice. The same intestinal inflammation was eventually observed in the recipient mice, verifying that *P. gingivalis*-induced intestinal dysbiosis is itself sufficient to promote intestinal inflammation (**Figure 2**).

### 3.1 Pathways of translocation of oral bacteria to the gut

More than half of the bacterial species in the gastrointestinal system undergo oral-gut translocation (Schmidt et al., 2019). First, the oral cavity and gut are inherently linked through saliva. Studies have demonstrated that periodontal bacteria migrating via enteral dissemination (salivary pathway) are able to colonize and survive in the gut for at least 24 hours (Bao et al., 2022). Second, the rich blood circulation in the oral cavity and the ulcerated surface of the lining of periodontal pockets in patients with periodontitis also allow the oral microbiota to colonize the gut through hematogenous dissemination. A study comparing colorectal cancer (CRC) colonization by gavage vs. intravenous inoculated *Fusobacterium nucleatum* in a mouse model and found that hematogenous fusobacteria were more successful in CRC colonization than gavaged ones. This may indicate that the circulatory system appears to be theefficient route for some periodontal bacteria to reach the gut. However, more evidence is still needed to support this.

**Figure 3:**
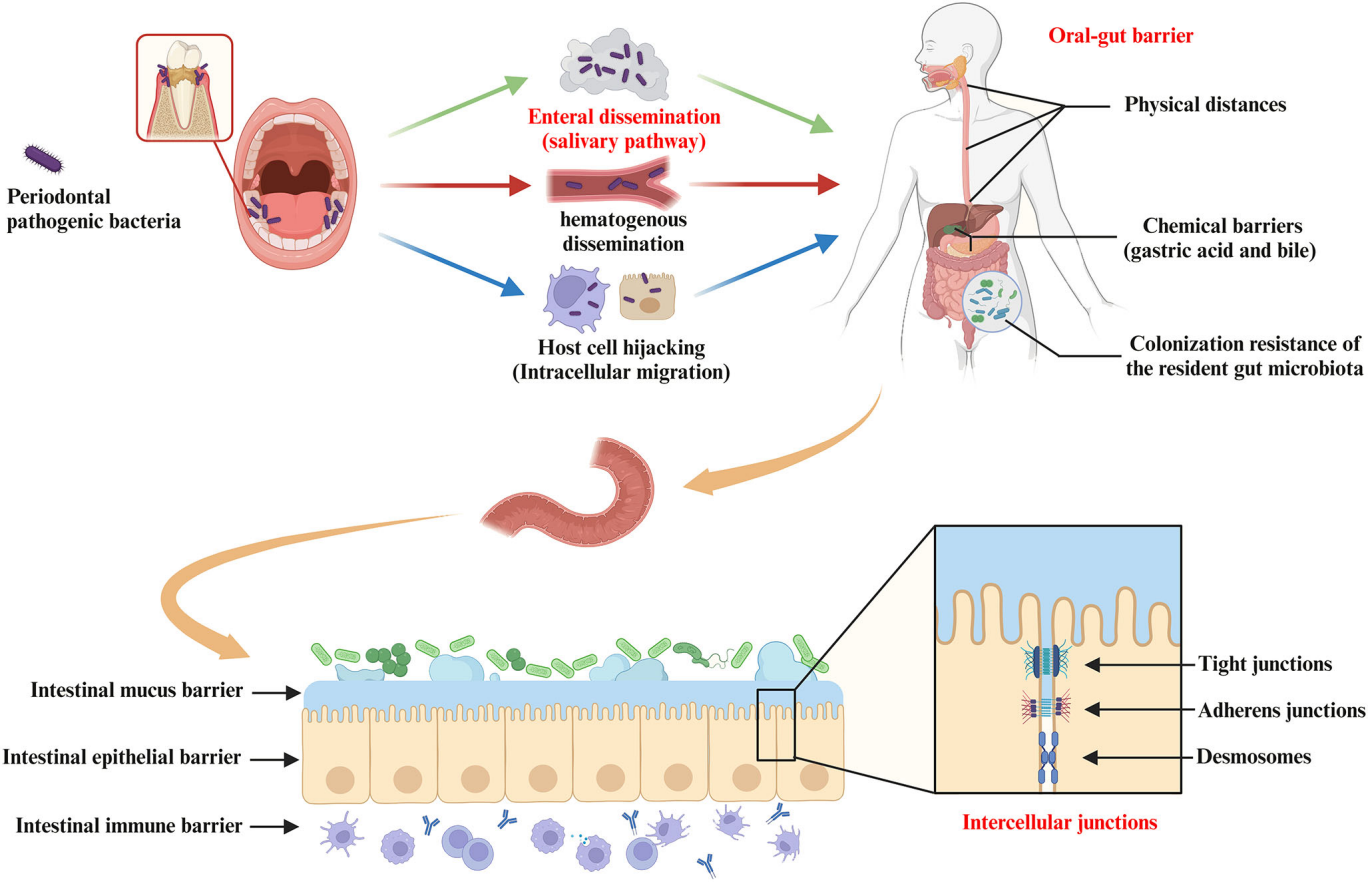
Pathways of oral bacterial translocation to the gut and host barrier systems limiting oral-gut microbial translocation. Intestinal translocation of periodontal pathogenic bacteria is pathogenically important in the progression of systemic diseases mediated by the oral-gut axis. Possible routes of oral microbiota translocation to the gut include (i) migration by enteral dissemination (salivary route), (ii) colonization of the gut by hematogenous dissemination, and (iii) hijacking of host cells as a vehicle for migration to the gut. First, the oral-gut barrier is one of the important strategies for the host to maintain microbial homeostasis in order to limit microbial translocation between the oral cavity and the gut. The oral-gut barrier includes physical distances and chemical barriers (e.g. gastric acid and bile), as well as colonization resistance of the resident gut microbiota to the intestinal migration of oral microbes with pathogenic potential. If oral bacteria break through the oral-gut barrier to reach the lumen, the barrier function of the intestinal wall is still able to separate the host from adjacent microbiota. The intestinal barrier consists of (i) the intestinal mucus barrier composed of mucin and antimicrobial peptides (AMPs) overlying IEC, (ii) the intestinal epithelial barrier composed of IEC and intercellular junctions (e.g. tight junctions, adherens junctions and desmosomes), and (iii) the intestinal immune barrier composed of innate and adaptive immune cells (gut-associated lymphoid tissue).

Finally, after invasion of host cells, periodontal pathogenic bacteria (e.g. *P. gingivalis* and *F. nucleatum*) may survive within them and subsequently disseminate to the gut (Xue et al., 2018; Kitamoto and Kamada, 2022a). For example, periodontal bacteria with virulence factors that inhibit phagolysosome formation are able to survive within the host cell and migrate intracellularly via Trojan horse mode or vesicular trafficking (Yilmaz et al., 2004; Sansores-España et al., 2021). *P. gingivalis* has been shown to survive within macrophages, epithelial cells, endothelial cells and smooth muscle cells and to spread from one cell to another (Wang et al., 2007; Li et al., 2008; Carrion et al., 2012). Thus, theoretically, periodontal bacteria may hijack these cells as a vehicle for migration to the gut (Kitamoto et al., 2020; Hajishengallis and Chavakis, 2021). However, more evidence is still required to support that periodontal bacteria translocated via this pathway are of sufficient pathogenic significance in the oral-gut axis mediating systemic disease progression. In conclusion, enteral dissemination (the main pathway), hematogenous dissemination, and host cell hijacking are all possible ways in which the oral microbiota can translocate to the gut, resulting in a complex association of the oral-gut axis based on microbial crosstalk (**Figure 3**).

### 3.2 Breach of the oral-gut barrier by periodontal bacteria

The oral-gut barrier is one of the important strategies for the host to maintain microbial homeostasis in order to limit microbial translocation between the oral cavity and the gut. The oral-gut barrier includes physical distances and chemical barriers (e.g. gastric acid and bile), as well as colonization resistance of the resident gut microbiota to the intestinal migration of oral microbes with pathogenic potential (**Figure 3**). Intestinal commensal bacteria can play an important role in regulating intestinal mucosal homeostasis and inhibiting colonization of potential oral pathogens via distinct mechanisms (Suárez et al., 2021). For example, intestinal commensal bacteria can compete for nutrients and produce antimicrobial peptides and metabolites that affect the colonization and virulence of oral pathogens. Intestinal commensal bacteria also promote the induction of effector T and B cells locally and systemically in response to pathogens (Suárez et al., 2021). However, this protective mechanism of colonization resistance may be disturbed by microbiota imbalance or local changes in host response, leading to disruption of host-microbiome homeostasis.

### 3.3 Effect of periodontal bacteria on intestinal barrier function

**Figure 4:**
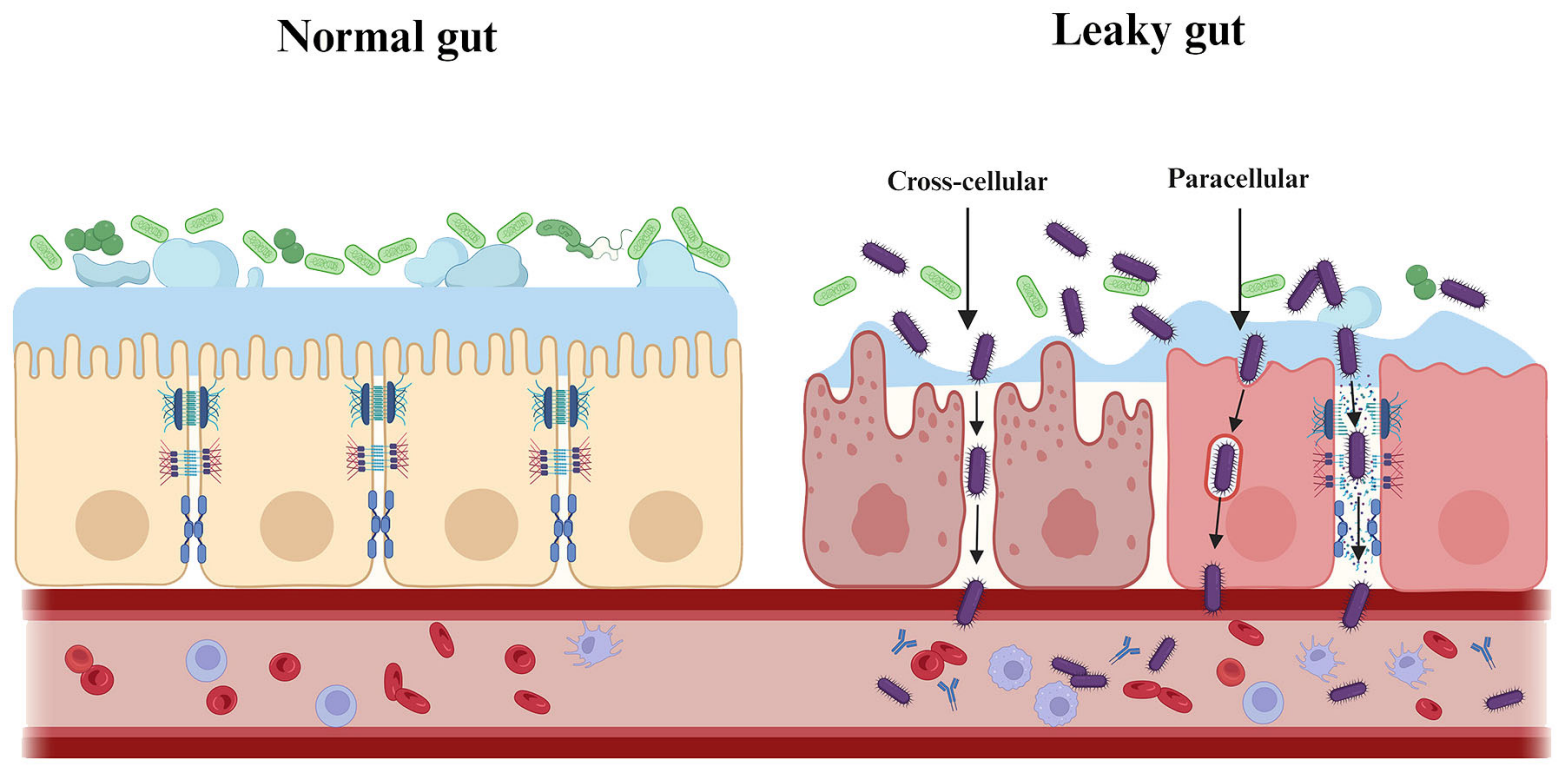
Periodontal pathogenic bacteria trigger and exacerbate “leaky gut syndrome”. Prolonged translocation of periodontal bacteria to the gut may further trigger and exacerbate the putative disease “leaky gut syndrome” by affecting gut microbiota homeostasis and barrier function. As shown, periodontal pathogenic bacteria are involved in the pathogenesis of various systemic diseases by either directly/indirectly reducing intestinal epithelial intercellular junctions (paracellular) or by invading intestinal epithelial cells (transcellular), which ultimately affects and breaches the intestinal barrier.

The intestinal barrier has several physical and chemical barriers separating the host from the adjacent microbiota (**Figure 3**). However, when the intestinal barrier function is pathologically altered, increased intestinal permeability and inflammatory response may allow the gut to “leak”, i.e. allowing pathogenic substances to cross the intestinal wall and spread systemically with pathological consequences (Hollander and Kaunitz, 2020). Prolonged translocation of periodontal bacteria to the gut may further trigger and exacerbate the putative disease “leaky gut syndrome” by affecting gut microbiota homeostasis and barrier function, thus participating in the pathogenesis of various gastrointestinal and systemic diseases (Kinashi and Hase, 2021) (**Figure 4**). This will be discussed in more detail below.

#### 3.3.1 Intestinal mucus barrier

A gel-like sieve structure formed by mucin overlying intestinal epithelial cells (IECs) is the first physical barrier that separates bacteria in the lumen from the IEC (Pelaseyed et al., 2014). At the same time, antimicrobial peptides (AMPs) (e.g. alpha-defensins, lysozyme C and C-type lectins), which are components of the intestinal mucus, form a chemical barrier that prevents and clears intestinal pathogens and protects intestinal cells from external factors (Birchenough et al., 2015; Liu et al., 2021b).

Periodontal bacteria are capable of disrupting the integrity and function of the intestinal mucus barrier. For example, *P. gingivalis* secretes Gingipain B (RgpB) that cleaves mucin 2 (MUC2) to disrupt its polymerization (van der Post et al., 2013). *P. gingivalis* can also circumvent or manipulate AMP to disrupt intestinal homeostasis, further exacerbating intestinal ‘leakiness’ (Ji et al., 2007; Hussain et al., 2015).

#### 3.3.2 Intestinal epithelial barrier

The intestinal epithelial barrier is provided by IEC that form multiple types of junctions, including tight junctions (TJs), adherens junctions and desmosomes (**Figure 3**). The composition and abundance of the different components of intercellular junctions are decisive for intestinal permeability and, together with IEC, maintain an important mechanical barrier preventing the transit (cross-cellular/paracellular) of pathogenic substances such as toxins and bacteria from the lumen to other parts of the body. In addition, IEC can regulate the persistence of antigens on the surface through epithelial shedding (Suárez et al., 2021).

In affecting and breaching the intestinal epithelial barrier, periodontal pathogenic bacteria can directly/indirectly reduce inter-epithelial adhesions, or invade and transit apically through epithelial cells in several ways. 1) Downregulation of adhesion molecule expression. Pathogenic bacteria (e.g. *P. gingivalis*, *A. actinomycetemcomitans* and *T. denticola*) reduce intestinal epithelial surface expression of E-cadherin, resulting in the destruction of adherens junctions and allowing transmigration (Devaux et al., 2019). In animal studies, a downregulation of tight junctions at the mRNA level was also observed in the gut of mice following oral administration of periodontal pathogenic bacteria (e.g. *P. gingivalis* and *F. nucleatum*) (Meilian et al., 2016; Olsen and Yamazaki, 2019; Cao et al., 2020). 2) Degradation of adhesion molecules. For example, *P. gingivalis* uses gingival proteases to directly degrade tight junctions (e.g. Occludin) and adherens junctions (E-cadherin) (Takahashi et al., 2019; Tsuzuno et al., 2021). 3) Cause re-distribution of E-cadherin from membrane to cytoplasm. Substantial remodeling of cell junctions may attenuate the integrity of the intestinal epithelial barrier. The recombinant cyto-lethal distending toxin (Cdt), a putative virulence factor of *A. actinomycetemcomitans*, affects epithelial barrier function by altering E-cadherin’s cytosolic distribution (Takahashi et al., 2019). 4) Excessive cell death may lead to barrier dysfunction and translocation of pathogenic bacteria (Crawford et al., 2022). *F. nucleatum* and its LPS have been shown to promote cell apoptosis and pro-inflammatory cytokine production in gut by activating autophagy pathway in IEC *in vivo* and *in vitro* (Su et al., 2020).

Finally, periodontal pathogenic bacteria indirectly reduce inter-epithelial adhesion by stimulation of inflammation. Mechanistically, *F. nucleatum* targets caspase activation and caspase recruitment domain 3 (CARD3) to activate the endoplasmic reticulum stress (ERS) pathway or the IL-17F/NF-κB pathway, mediating damage to the intestinal epithelial barrier (Cao et al., 2020; Chen et al., 2020a). *F. nucleatum* can also increase inflammatory genes (e.g. NF-κB) through its FadA adhesin binding to E-cadherin (Pignatelli et al., 2023). In addition, studies have shown that even a single oral dose of *P. gingivalis* can cause the prevalence of inflammatory microbiota in the gut (Nakajima et al., 2015). The increase in the proportion of intestinal pathogenic bacteria leads to the enrichment of LPS in the lumen. LPS is an important stimulus for impaired intestinal epithelial barrier function via an intracellular mechanism involving TLR-4-dependent up-regulation of CD14 membrane expression (Ciesielska et al., 2021).

#### 3.3.3 Intestinal immune barrier

As the largest immune organ in the body, the intestinal immune barrier consists of innate and adaptive immune cells (gut-associated lymphoid tissue). Disruption of the homeostasis of the gut microbiota and immune barrier may trigger an excessive intestinal immune response, leading to the intestinal barrier damage and ultimately inducing systemic diseases associated with altered intestinal immune responses (Liu et al., 2021b).

Studies have observed an upregulation of pro-inflammatory cytokines in the gut by periodontal pathogenic bacteria. Colonization by *P. gingivalis* drives an increase in pro-inflammatory factors such as IL-6 and TNF-α in the gut (Lee et al., 2022). *P. gingivalis* also induces an increase in intestinal-derived IL-17 in peripheral blood (leaky gut) and has been shown to be associated with increased LPS in the gut (Arimatsu et al., 2014; Sato et al., 2017). The exact mechanism of action currently requires further research to elucidate.

Disruption of the Th17/Treg balance and increased secretion of pro-inflammatory cytokines in the body are important ways in which periodontal pathogenic bacteria can trigger systemic disease by affecting immune homeostasis in the gut. Periodontal pathogenic bacteria in the gut have been shown to be involved in the pathogenesis of diseases including inflammatory bowel disease (IBD), rheumatoid arthritis (RA), Parkinson’s disease (PD), psoriasis, and liver disease through activation of Th17-related pathways (Vernal et al., 2014; Bunte and Beikler, 2019; Feng et al., 2020; Kitamoto et al., 2020; Liu et al., 2021a). It has also been found that recovery of the Th17/Treg balance in periodontitis by the local injection of 3D-exos (a mesenchymal stem cell-derived exosomes, MSC-exos) attenuated experimental colitis. However, further human studies to validate the efficacy and feasibility of Th17-targeted therapies are still lacking.

## 4 Impact of periodontal bacteria on systemic diseases via the oral-gut axis

### 4.1 Intestinal diseases

#### 4.1.1 Inflammatory bowel disease

IBD is a chronic, recurrent inflammatory disease of the GIT. Periodontitis, also a chronic inflammatory disease, significantly increase the risk of IBD and its disease severity (Vavricka et al., 2013; Lin et al., 2018). Increased periodontal pathogenic bacteria in the gut of IBD patients is positively correlated with the severity of IBD (Lucas López et al., 2017; Schirmer et al., 2018). Animal studies suggest that colitis may be worsened by gut microbial disturbances, which was promoted by gavage of periodontitis salivary microbiota or by infection with periodontal pathogenic bacteria (e.g. *P. gingivalis* and *F. nucleatum*) (Cao et al., 2020; Tsuzuno et al., 2021). Successful intestinal colonization of those inoculated oral pathobionts serves as an important trigger to exacerbate IBD. Further study on intestinal colonization, Kitamoto et al. found that a significant level of gut colonization by oral pathobionts was only observed in mice with both oral and gut dysbiosis, rather than only one of the two (Kitamoto and Kamada, 2022b). This implied that periodontal bacteria are pathogenic only to susceptible hosts or promote the progression of pre-existing IBD. Possible pathogenic mechanisms for IBD after intestinal colonization include the ability of periodontal bacteria to compromise the intestinal barrier (downregulation of TJs), enhance intestinal immune responses (e.g. induction of M2 macrophage polarization), and affect intestinal metabolism (e.g. decreased unsaturated fatty acid synthesis and increased arachidonic acid metabolism). Recent evidence suggested that *P. gingivalis* administration aggravates IBD via a gut microbiota-metabolite linoleic acid (LA)-Th17/Treg cell balance axis. Mechanistically, under Th17-polarizing culture conditions, LA, by specifically binding to AHR as an antagonist, drive Stat1 phosphorylation at Ser727, which in turn represses IL-17 while enhancing Foxp3 expression. Further studies revealed that LA supplementation alleviates *P. gingivalis*-induced colitis and exacerbation of Th17/Treg cell imbalance (Jia et al., 2024).

#### 4.1.2 Colorectal cancer

CRC has the third highest incidence of all cancers (Bray et al., 2018). The gut microbiota of CRC patients is significantly altered compared to that of the healthy population (Chen et al., 2012). Patients with more severe periodontitis have a higher number of oral-derived microorganisms in their gut microbiota and a higher risk of developing CRC, as well as a worse prognosis (Momen-Heravi et al., 2017; Negrut et al., 2023).

In addition to the increased number of oral pathobionts, analysis using 16S rRNA gene sequencing revealed that some of the periodontal bacteria (e.g. *F. nucleatum*, *P. gingivalis*, *T. denticola*, and *P. intermedia*) were significantly associated with the progression of CRC (Negrut et al., 2023). Animal studies further transplanted the fecal microbiota of periodontitis patients into CRC mice and the same tumor-promoting effect was observed, suggesting that periodontal bacteria may promote CRC by remodelling the oral and gut microbiota (Shi et al., 2023). The current mechanistic study found that in a dysregulated intestinal environment mediated by periodontal bacteria, opportunistic pathogens are able to exploit tumor surface barrier defects, invade normal colonic tissue and induce local inflammation, while producing genotoxic metabolites to induce oncogenic transformation of colonic epithelial cells (Chen et al., 2017).

Many periodontal bacteria are associated with the development of gastrointestinal cancers, with F. *nucleatum* being most closely associated with CRC (Fan et al., 2018; Brennan and Garrett, 2019). *F. nucleatum* is usually detected in cancer tissue of CRC patients as well as in secondary distal metastases (e.g. liver and lung) (Flanagan et al., 2014; Abed et al., 2016; Mima et al., 2016; Bullman et al., 2017). Moreover, patients with higher abundance of *F. nucleatum* in the cancer tissue usually have a shorter survival time and are more likely to recur (Wang et al., 2021). *F. nucleatum* can be involved in influencing the various stages of CRC development through a variety of mechanisms.

At the stage of tumor progression, *F. nucleatum* acts to induce a pro-cancer immune microenvironment and assists tumor immune evasion by inhibiting anti-tumor cells as well as increasing the number and function of immunosuppressive cells. For example, *F. nucleatum* binds and activates the human inhibitory receptors TIGIT and CEACAM1, thereby inhibiting T and NK cells and suppressing anti-tumor immunity (Gur et al., 2015). *F. nucleatum* also suppresses immunity and increases tumor multiplicity by selectively recruiting tumor-infiltrating myeloid cells (Kostic et al., 2013). In addition to modulating immune cells, *F. nucleatum* can target tumor cells themselves. *F. nucleatum* can increase the expression of inflammatory genes (e.g. NF-κB) and oncogenes (e.g. Myc and Cyclin D1) via the FadA/E-cadherin/β-catenin pathway (Rubinstein et al., 2013). This active pathway also leads to *F. nucleatum*-induced over-expression of chk2, which facilitates DNA damage and tumor growth (Guo et al., 2020). Furthermore, LPS produced by *F. nucleatum* can upregulate microRNA-21 expression via the TLR4/Myd88/NF-κB pathway, thereby activating the MAPK pathway and enhancing cancer cell proliferation (Yang et al., 2017). In promoting tumor metastasis, *F. nucleatum* can activate autophagy signaling via the upregulation of CARD3 expression, regulate epithelial-mesenchymal transition (EMT), activate the NF-κB pathway and transmit exosomes (Chen et al., 2020b; Chen S. et al., 2020; Guo et al., 2021; Kong et al., 2021).

During the tumor treatment phase, *F. nucleatum* increases chemotherapy resistance in CRC patients, ultimately leading to tumor recurrence. *F. nucleatum* reduces the responsiveness of CRC cells to chemotherapeutic agents (e.g. oxaliplatin and 5-fluorouracil) by upregulating BIRC3 via the TLR4/NF-κB pathway or by inhibiting specific miRNAs involved in autophagy (Yu et al., 2017; Zhang et al., 2019). Therefore, *F. nucleatum* has been used as a non-invasive biomarker for CRC screening and assessment of prognosis.

Finally, *F. nucleatum*-based bacteriotherapy may be a potential therapeutic target for CRC. *F. nucleatum* expresses a variety of virulence factors associated with adhesion (e.g. RadD, Aid1 and Fap2) and invasion (e.g. FadA) of host cells, thereby exerting its pathogenicity through colonization, dissemination, evasion of host defenses and induction of host responses. Some of the *F. nucleatum* virulence factors have been shown to be closely associated with CRC. For example, FadA promotes the growth of CRC cells, while Fap2 enhances CRC progression by suppressing immune cell activity (Shang and Liu, 2018). An *F. nucleatum*-specific bacteriophage, FNU1, was found to kill cells and eradicate onco-bacterium from tumor tissue (Wang et al., 2024). In addition, antibiotic inoculation with *F. nucleatum* could eliminate *F. nucleatum* from breast cancer and further suppressed *F. nucleatum*-induced tumor growth (Parhi et al., 2020). Therefore, *F. nucleatum* (or other oral bacteria)-mediated therapies for CRC may be worth exploring further.

### 4.2 Rheumatoid arthritis

RA is a chronic inflammatory autoimmune disease characterized by bone destruction in multiple joints throughout the body. RA patients often have significant alterations in their oral and gut microbiota that correlate with the severity of joint destruction and the efficacy of antirheumatic treatment in RA patients (Phillips, 2015; Zhang X. et al., 2015; Drago et al., 2019; Möller et al., 2020). Several periodontal bacteria (e.g. *P. gingivalis* and *Prevotella intermedia*) are detected in the gut, serum and synovial fluid of RA patients (Martinez-Martinez et al., 2009; Pianta et al., 2017; Drago et al., 2019). RA may be a reactive arthritis aggravated by the repeated translocation of these periodontal bacteria to the joints. Whether oral pathobionts reach the joints through the oral-gut axis or through hematogenous dissemination, most studies suggest that both routes are possible, but more research is needed to determine which route is predominant or prior.

As the most important diagnostic biomarker for RA, serum levels of anti-citrullinated protein antibodies (ACPA) positively correlate with the severity of both RA and periodontitis (Lappin et al., 2013; Bellando-Randone et al., 2021). *P. gingivalis* can convert arginine residues in proteins to citrulline and increases serum levels of ACPA (Möller et al., 2020). *A. actinomycetemcomitans* can also indirectly increase protein citrullination through the pathway of leukotoxin (LtxA)-induced peptidyl-arginine deiminase (PAD) dysregulation, thereby increasing ACPA levels (Konig et al., 2016). Thus, one of the important mechanisms by which periodontal bacteria promote RA via the oral-gut axis is chronic exposure to citrullinated protein (CP) triggers the expression of ACPA in joint synovium throughout the body.

In an arthritis mouse model, oral administration of *P. gingivalis* induced dysbiosis of the gut microbiota, increased IL-6 and CP production in serum, joint and intestinal tissues, and exacerbated joint destruction (Hamamoto et al., 2020). Further, fecal microbiota transplantation (FMT) from *P. gingivali*-inoculated experimental arthritis mice reproduced donor gut microbiota and resulted in severe joint destruction with increased IL-6 and CP production in joint and intestinal tissues. This suggests that the gut dysbiosis induced by *P. gingivalis* oral infection may be sufficient to trigger or exacerbate arthritis.

*F. nucleatum* is also enriched in the gut of RA patients and positively correlate with RA severity (Hong et al., 2023). Mechanistically, *F. nucleatum* delivers FadA to the joints via outer membrane vesicles (OMVs) and triggers synovial inflammation by activating the Rab5a-YB-1 axis in synovial macrophages. In addition, some studies have treated with the zonulin antagonist larazotide acetate, which specifically increases intestinal barrier integrity (modulating TJs), effectively reduces arthritis onset (Tajik et al., 2020).

The mechanism by which periodontal bacteria promote RA may also be related to the activation of Th17-related pathways. Furthermore, periodontal bacteria (e.g. *P. gingivalis* and *Prevotella nigrescens*) are involved in inflammatory bone destruction in RA by increasing the intestinal hyperimmune response triggered by Th17 cellular immune responses, which can induce higher levels of RANKL and TRAP^+^ osteoclasts (Vernal et al., 2014). *P. nigrescens* also can reduce the osteoprotective effects of Th2 (de Aquino et al., 2014). All of this evidence demonstrates that the oral-gut axis plays an important role in the influence of periodontal bacteria on RA.

### 4.3 Brain diseases

**Figure 5:**
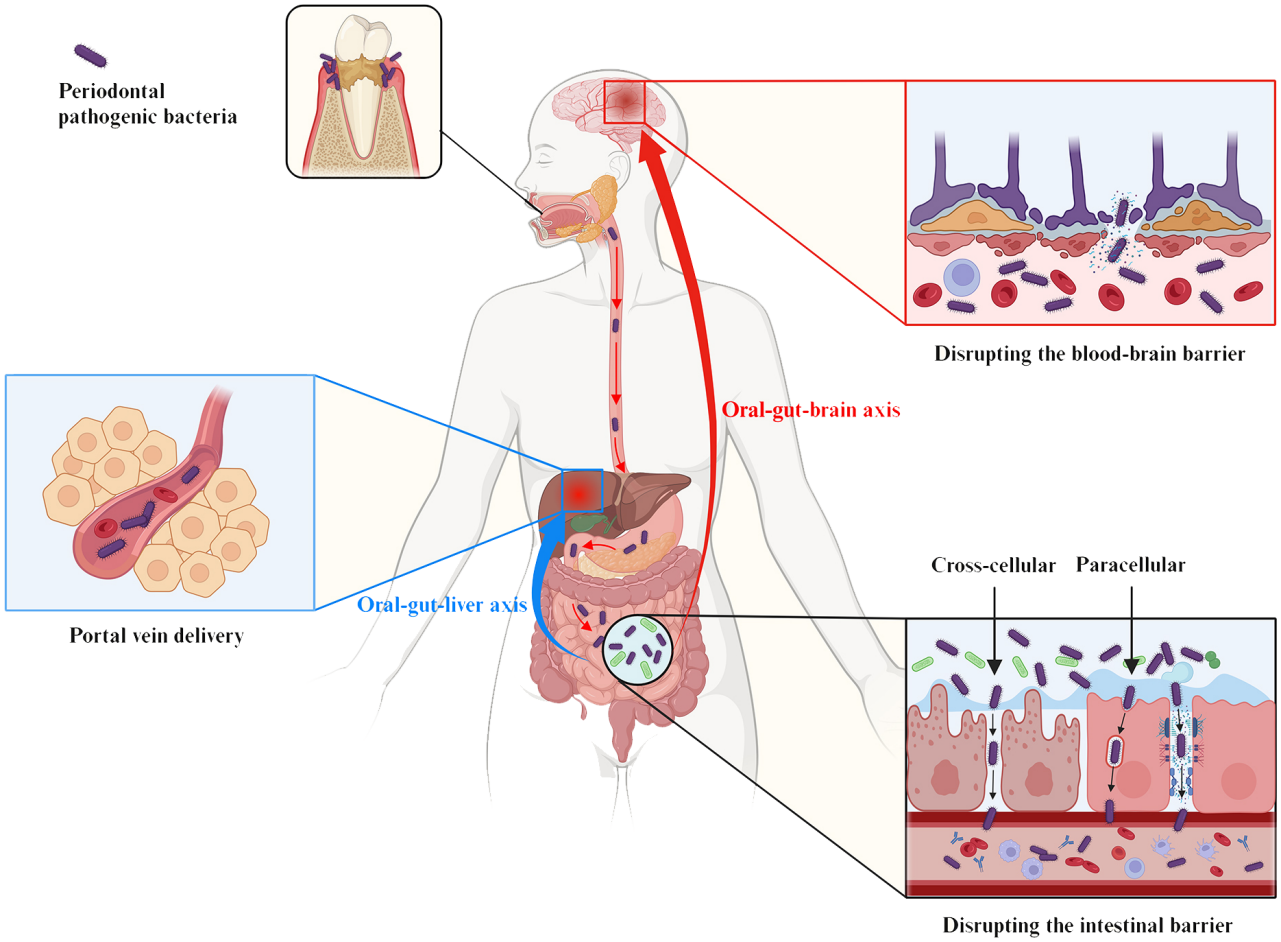
The oral-gut-liver axis and the oral-gut-brain axis serve as the pathophysiologic basis for the influence of periodontal pathogenic bacteria on liver and brain diseases via the intestinal pathway. The gut establishes an anatomical dependence on the liver via portal circulation for the direct delivery of pathogens or metabolites to the liver, a pathophysiologic pathway termed the “oral-gut-hepatic axis” (blue symbols). In addition, the “oral-gut-brain axis” (red symbols) refers to periodontal pathogenic bacteria that trigger neuroinflammatory and pathological changes in the brain based on the disruption of the intestinal barrier and the BBB. As shown, periodontal pathogenic bacteria are involved in the pathogenesis of various systemic diseases by either directly/indirectly reducing intestinal epithelial intercellular junctions (paracellular) or by invading IEC (transcellular), which ultimately affects and breaches the intestinal barrier.

Periodontal bacteria may be indirectly associated with brain diseases through the gut pathway. In addition to ischemic (e.g. stroke) and autoimmune (e.g. multiple sclerosis) brain diseases, evasion strategies of specific pathogens may also alter the function of the blood-brain barrier (BBB) (Daneman and Rescigno, 2009). Here we focus on the pathogenic mechanisms of the oral-gut-brain axis in brain disease, i.e., periodontal bacteria disrupt the gut barrier and BBB, further triggering neuroinflammatory and pathological changes in the brain (**Figure 5**) (Sansores-España et al., 2021).

#### 4.3.1 Alzheimer’s disease

AD is the most common neurodegenerative disease, with progressive behavioral and cognitive impairment as the main clinical feature. Endothelial cells protecting the BBB have been shown to be infected by periodontal bacteria, and virulence factors such as *P. gingivalis-*LPS and gingipains can even be detected directly in the brains of AD patients (Dominy et al., 2019). Animal studies provide evidence to support this view. Mice with experimental periodontitis showed significant dysbiosis of the gut microbiota, disruption of the intestinal barrier and BBB, and increased levels of LPS in the serum and brain. Meanwhile, neuropathological alterations, including neuronal loss, synaptic injury, and glial activation, as well as progressive cognitive deficits were also observed (Xue et al., 2020).

The pathological mechanisms by which periodontal bacteria promote AD may include the following three aspects. First, periodontal bacteria trigger an excessive accumulation of Aβ in the brain. *P. gingivalis* may increase Aβ production by releasing gingipains-rich OMVs that drive NLRP3 inflammasome activation, ASC speck aggregation and pyroptotic cell death (Fleetwood et al., 2017; Dominy et al., 2019). Upregulation of advanced glycation end products (RAGE) expression in brain endothelial cells also mediates Aβ influx following *P. gingivalis* infection (Zeng et al., 2021). In addition, Intestinal-derived LPS, including *P. gingivalis*-LPS, has been shown to be abundant in the AD brain and has been found to be associated with Aβ plaque formation through activation of the TLR4-mediated NF-κB and MAPK pathways (Zhao et al., 2017).

Second, periodontal bacteria mediate the formation of NFTs in neuronal cells and promote AD (Arnsten et al., 2021). Formation of NFTs results from hyperphosphorylation of the tau protein (Narengaowa et al., 2021). Gingipains can cause proteolysis of tau (tau phosphorylation and cleavage) and subsequent NFTs formation by activating caspase-3. *P. gingivalis* and its virulence factors, such as lysine-gingipain (Kgp) and arginine-gingipain B (RgpB) are independently and positively correlated with tau load in AD brains (Dominy et al., 2019).

Finally, neuronal necrosis due to neuroinflammation is also a key mechanism for AD. Periodontal bacteria and their virulence factors may activate and accumulate large numbers of microglia through the gut, inducing synaptic toxicity and neuronal death, ultimately exacerbating neurodegeneration (Narengaowa et al., 2021). For example, intraneuronal gingipains may drive neuronal NLRP1 activation, resulting in pyroptosis of neurons and activation of caspase-1, leading to release of the neuroinflammatory interleukins IL-1β and IL-18 (Dominy et al., 2019).

Therapeutically, administration of small-molecule inhibitors of gingipain attenuates neurodegeneration and significantly decreases the host Aβ response to *P. gingivalis* brain infection, thereby slowing or preventing further accumulation of pathology in AD patients (Dominy et al., 2019). Another study has found a favorable effect of periodontal treatment on AD-related brain atrophy. However, more research is still needed to explore the relationship between periodontal treatment and AD (Schwahn et al., 2022).

#### 4.3.2 Parkinson’s disease

Intestinal inflammation and leakage promoted by oral administration of periodontal bacteria (e.g. *P. gingivalis*) may lead to significant loss of dopaminergic neurons and microglial activation in the SNpc, thereby triggering PD (Feng et al., 2020). In animal studies, FMT treatment ameliorated gastrointestinal dysfunction and motor deficits in PD mice by restoring intestinal homeostasis, attenuating damage to the intestinal barrier and BBB and suppressing neuroinflammation and dopaminergic neuronal damage in the substantia nigra (SN) (Zhao Z. et al., 2021). Among the human studies, a recent double-blind, placebo-controlled, randomized trial also suggested that a single FMT induced mild, but long-lasting beneficial effects on motor symptoms in patients with early-stage PD (Bruggeman et al., 2024).

α-synuclein is thought to be a central molecular player involved in the pathogenesis of PD through the oral-gut-brain axis. An endoscopic biopsy study showed a significant correlation between the level of α-synuclein accumulation in neurites of the enteric nervous system and the degree of inflammation of intestinal wall (Stolzenberg et al., 2017). Periodontal bacteria may cross the IEC and induce misfolding and aggregation of α-synuclein in specific enteric neurons. The aggregated α-synuclein will then migrate to the brain via the vagus nerve (Holmqvist et al., 2014; Garrido-Gil et al., 2018). *P. gingivalis*-mediated activation of leucine-rich repeat kinase 2 (LRRK2) has been shown to consistently induces α-synuclein expression in the gut of R1441G mice, triggering neuroinflammation and subsequent degeneration of dopaminergic neurons (Kozina et al., 2018; Feng et al., 2020).

In addition, IL-17A may also be involved in the effect of periodontal bacteria on PD via the gut (Huang et al., 2014). For example, oral administration of *P. gingivalis* leads to IL-17A immunoreactivity in the peripheral system and upregulates IL-17RA protein levels in dopaminergic neurons (Feng et al., 2020). Peripheral IL-17A crosses the BBB and mediates dopaminergic neuron degeneration via IL-17RA in microglia (Liu Z. et al., 2019).

#### 4.3.3 Autism spectrum disorder

Autism spectrum disorder (ASD) is a neurodevelopmental disorder characterized by abnormal language and interaction skills and repetitive stereotyped behavior (Qiao et al., 2022). Studies have observed disruption of the intestinal barrier and BBB in post-mortem brain tissue and gut in ASD subjects, as well as significant neuroinflammation in the brain (Fiorentino et al., 2016). ASD patients have been further found to have unique oral and gut microbiota distribution patterns, including elevated F/B ratio and increased abundance of opportunistic pathogens, and this bacterial community differences are positively correlated with ASD severity (Tomova et al., 2015; Kong et al., 2019; Johnson et al., 2020). A study that transferred the oral microbiota of ASD donors to an antibiotic-mediated microbiota-depleted mouse model found the induction of ASD-like behaviors (e.g. impaired social behavior) (Kong et al., 2019; Qiao et al., 2022). Significant differences in oral and gut microbiota structure and altered neurosignaling activities, including upregulation of serotonin-related gene expression and TGF-β signaling pathway, were observed in ASD microbiota recipient mice compared to typical development microbiota recipient mice. Furthermore, increased serotonin-related gene expression in ASD microbiota recipient mice was associated with both autistic behaviors and changes in abundance of specific oral microbiota, such as *Porphyromonas* spp. These evidences highlight the important influence of the oral microbiota in the gut-brain connection.

In addition to these, a variety of other brain disorders, including neuropsychiatric disorders (e.g. depression), neurodegenerative diseases (e.g. multiple sclerosis) and cerebrovascular diseases (e.g. ischemic stroke), may also be associated with the mediating effects of periodontal bacteria via the oral-gut-brain axis pathway (Zhang Z. et al., 2015; Cunha et al., 2019; Maitre et al., 2020; Brown et al., 2021). For example, in a rat model study, oral gavages with *P. gingivalis* and *F. nucleatum* simultaneously induced periodontitis, neuroinflammation and depression-like behavior (Martínez et al., 2021). This study further identified *F. nucleatum* in the frontal cortex of experimental rats and also demonstrated that gastrointestinal translocation of periodontal bacteria can lead to the entry of peripheral LPS into the rat brain and elicit TLR-4-dependent neuroinflammation by finding evidence for the existence of an APOA1-mediated transport mechanism (Martínez et al., 2021). However, clinical studies are still needed for further confirmation.

### 4.4 Liver diseases

The gut microbiota may transport bacteria or metabolites directly to the liver via portal circulation (Hou et al., 2022). The impaired gastric acid and bile secretion prevalent in chronic liver disease (e.g. cirrhosis) may also make the gut more susceptible to translocation and colonization by oral bacteria (e.g. *P. gingivalis* and *T. denticola*) (Kakiyama et al., 2014; Qin et al., 2014; Wang et al., 2022). The anatomical dependence of the liver on the gut through metabolic exchange and pathogen translocation is therefore the basis for the pathophysiology of periodontal bacteria affecting liver disease via the gut pathway, the “oral-gut-liver axis” referred to in many current studies (**Figure 5**).

Epidemiological evidence suggests that having severe periodontitis increases the prevalence of non-alcoholic fatty liver disease (NAFLD), which recently renamed Metabolic dysfunction-associated fatty liver disease (MAFLD), mortality associated with cirrhosis, and progression of NAFLD towards fibrotic liver injuries (Akinkugbe et al., 2017; Ladegaard Grønkjær et al., 2018). In addition, patients with hepatocellular carcinoma in combination with periodontitis tend to have worse cancer stage, liver function and prognosis (Tamaki et al., 2011). Periodontitis may affect liver disease by modulating the oral and gut microbiota.

Dysregulated oral and gut microbiota may act as important mediators between periodontal bacteria and liver disease. Oral administration of periodontal bacteria (e.g. *P. gingivalis* and *A. actinomycetemcomitans*) may increase the risk of liver disease by altering the F/B ratio of the gut microbiota and increasing intestinal leakage (regulating TJs) and serum endotoxin levels (Arimatsu et al., 2014; Nakajima et al., 2015; Komazaki et al., 2017; Åberg and Helenius-Hietala, 2022). Dysregulated gut microbiota can also affect chronic liver disease by regulating bile acid metabolism and the production of short-chain fatty acids (SCFAs), ethanol and choline. Significant alterations in the composition of the gut microbiomes are present in patients with liver disease, including NAFLD, non-alcoholic steatohepatitis (NASH), cirrhosis and alcoholic liver disease (ALD). In contrast, FMT treatment can prevent NASH, ALD, hepatic encephalopathy (HE), and acute liver failure (ALF) in animal models by restoring intestinal homeostasis (Ferrere et al., 2017; Wang et al., 2017; Zhou et al., 2017; Liu et al., 2021a). Furthermore, genetic tracking revealed that the majority of species causing alterations in the gut microbiota of patients with liver disease are of buccal origin (Qin et al., 2014). Supporting this view, it has been shown that systemic periodontal therapy in cirrhotic patients modulates salivary and fecal microbiota dysbiosis, improves endotoxemia, as well as systemic and local inflammation (reduces inflammatory mediators), and improves quality of life and cognition in patients with HE (Bajaj et al., 2018). Periodontal treatment also improves biochemical markers (e.g. ALT and AST) in patients with NAFLD and cirrhosis (Yoneda et al., 2012; Komazaki et al., 2017; Nakahara et al., 2018). However, whether periodontal therapy improves organic liver changes remains to be investigated.

Periodontal bacteria and their virulence factors may reach the liver via the damaged gut and act directly on hepatocytes, Kupffer cells and hepatic stellate cells (HSCs) through activation of the PRR, hence triggering downstream pro-inflammatory cascades and ultimately affecting chronic liver disease (Schroeder and Bäckhed, 2016).

Periodontal treatment has been proposed as a potential approach to improve liver disease. It has also been suggested by some that the simultaneous treatment of periodontitis and intestinal disease may have a synergistic effect on patients with liver disease (Kuraji et al., 2023). That is, treatment of intestinal inflammation and intestinal ecological dysbiosis may prevent ectopic intestinal colonization caused by oral pathology, even in the presence of periodontitis. However, more studies are still needed to confirm these findings.

### 4.5 Obesity

Obesity is a multifactorial chronic inflammatory disease characterized by the overgrowth of adipose tissue. The degree of obesity (e.g. body mass index) has been found to be positively correlated with the degree of periodontal inflammation (e.g. periodontal inflamed surface area index and the level of periodontal bacteria in the oral cavity) (Chaffee and Weston, 2010; Nascimento et al., 2015; Aoyama et al., 2021).

Obesity patients have been shown to have alterations in both oral and gut microbiota (Wu et al., 2018; Benahmed et al., 2021). Microorganisms play an essential role in digestion, absorption, metabolism, energy use and immune regulation in the body (Turnbaugh et al., 2008). Just as germ-free (GF) mice were protected against high fat diet (HFD) induced obesity, whereas receiving FMT from obese mice increases fat deposition and metabolic disturbances in GF mice (Rabot et al., 2010). Oral administration of periodontal bacteria (e.g. *P. gingivalis* and *A. actinomycetemcomitans*) significantly alters the F/B ratio in the gut microbiota, which in turn may induce insulin resistance, hepatic steatosis and macrophage infiltration in adipose tissue, and promote further increases in body weight and adipose tissue in diet-induced obese mice (Arimatsu et al., 2014; Nakajima et al., 2015; Komazaki et al., 2017; Rojas et al., 2021).

The triggering of endotoxemia via the leaky gut is one of the risk factors for periodontal bacteria affecting obesity. In a mouse model, *P. gingivalis* significantly elevated serum endotoxin levels after only 1 hour of single oral administration, which in turn activated pro-inflammatory genes in the adipose tissue, blood vessels and liver of mice, ultimately increasing the risk of insulin resistance, atherosclerosis (AS) and NAFLD, respectively (Arimatsu et al., 2014). Recent studies suggest that *P. gingivalis*-induced endotoxemia may affect obesity by altering endocrine functions in brown adipose tissue (BAT) in mice, including glucose homeostasis, insulin sensitivity, and thermogenesis (Stanford et al., 2012; Hatasa et al., 2021). In addition, *P. gingivalis* administration may increase inflammation-related mRNA expression in BAT, such as TNF-α and IL6, and downregulate the expression of genes related to lipolysis and metabolism in BAT, such as *Lipe* and *Pnpla2* (Hatasa et al., 2021).

The chronic low inflammatory state caused by periodontal bacteria via the oral-gut axis promotes increased expression of pro-inflammatory adipokines (e.g. resistin) and decreased expression of anti-inflammatory adipokines (e.g. adiponectin) (Zimmermann et al., 2013). In contrast, periodontal treatment significantly reduced resistin levels and increased adiponectin levels in obesity patients (Bharti et al., 2013; Akram et al., 2017). However, there is no further evidence that periodontal therapy improves obesity by affecting adipokine secretion via the intestinal pathway.

### 4.6 Diabetes mellitus

DM is a group of metabolic diseases characterized by hyperglycemia caused by abnormal insulin secretion and/or action (Cho et al., 2018). A systematic review confirmed that severe periodontitis increased the prevalence of type 2 Diabetes mellitus (T2DM) by 53% (Wu et al., 2020). Periodontal patients with established DM tend to have poorer glycemic control and higher prevalence of DM-related complications and all-cause mortality (Sharma et al., 2016; Graziani et al., 2018; Ziukaite et al., 2018; Genco et al., 2020; Nguyen et al., 2020; Wu et al., 2020; Song et al., 2021). Periodontal treatment also facilitates effective glycemic management in DM patients (Chang et al., 2020).

The increased susceptibility of DM patients to certain infections due to altered innate immune response is thought to facilitate the systemic (including intestinal) dissemination of periodontal bacteria in the case of periodontitis (Toniolo et al., 2019; Barutta et al., 2022). The presence of *P. gingivalis* has been detected in fecal samples from DM mice following oral administration of *P. gingivalis* (Kashiwagi et al., 2021). DM Patients with periodontitis have significant alterations in both oral and gut microbiota, systemic pro-inflammatory cytokines and metabolic parameters (Li et al., 2020; Barutta et al., 2022; Silva et al., 2022). Studies have demonstrated that oral administration of periodontal bacteria (*P. gingivalis* and *P. intermedia*) induces altered gut microbiota and leaky gut prior to the development of systemic inflammation (Yamazaki et al., 2021).

The disruption of intestinal homeostasis mediated by oral administration of periodontal bacteria (e.g. *P. gingivalis* and *A. actinomycetemcomitans*) was confirmed by animal studies to induce systemic inflammation, metabolic changes and hepatic fat deposition in non-diabetic mice, while exacerbating fasting and postprandial hyperglycemia in DM mice (Arimatsu et al., 2014; Blasco-Baque et al., 2017; Komazaki et al., 2017; Kato et al., 2018; Sasaki et al., 2018; Kashiwagi et al., 2021). That is, in the pathogenic mechanism of periodontitis for DM, intestinal transmission of periodontal bacteria may induce/exacerbate insulin resistance and glucose intolerance by mediating entero-hepatic metabolic derangements. In addition, in a ligature-induced periodontitis mouse model, fasting blood glucose (FBG), serum glycated hemoglobin (HbA1c) and glucose intolerance levels were higher in the periodontitis group than in the control group (Li et al., 2021). In contrast, FBG, HbA1c, glucose tolerance levels and systemic inflammatory load were reversed in mice after elimination of periodontitis or depletion of the gut microbiota with antibiotics (Li et al., 2021). This further suggests that the gut microbiota may mediate the influence of periodontal bacteria on DM.

Firstly, intestinal transmission of periodontal bacteria leads to reduced diversity and altered F/B ratio in the intestinal microbiota. Individuals with low diversity of gut microbiota tend to exhibit more pronounced insulin resistance, hyperinsulinemia and increased susceptibility to DM (Cotillard et al., 2013; Le Chatelier et al., 2013). There are also mechanistic drivers for the correlation between taxa representation (*Bacteroidetes* and *Firmicutes*) of the gut microbiota and glycemic control (Larsen et al., 2010; Napolitano et al., 2014). For example, metformin is known to be a first-line treatment in patients with T2DM. Influencing bile acid metabolism and entero-endocrine hormone secretion by altering the gut microbiota is one of the important pharmacological mechanisms of metformin in the treatment of T2DM (Napolitano et al., 2014). Further studies found that serum concentrations of cholic acid and conjugates in T2DM patients were positively correlated with the microbiota abundance of Firmicutes and negatively correlated with Bacteroidetes (Napolitano et al., 2014). In addition, the F/B ratio of the gut microbiota was significantly correlated with circulating concentrations of peptide tyrosine-tyrosine (PYY) in serum (Napolitano et al., 2014). PYY, an intestinal hormone, has been shown to restore normal glucose regulation of insulin and glucagon secretion in DM rats (Ramracheya et al., 2016).

Secondly, alterations in gut metabolites mediated by periodontal bacteria may also contribute to the development of DM. In animal models, the presence of periodontitis or oral administration of *P. gingivalis* can disrupt the gut microbiota and serum metabolite profiles, and increase fasting hyperglycemia and entero-hepatic metabolic derangements associated with altered gut metabolism in DM mice (Kato et al., 2018; Kashiwagi et al., 2021). SCFAs are known to play an important role in mediating glucose metabolism and insulin sensitivity through multiple signaling pathways (Psichas et al., 2015). It was found that increased serum HbA1c levels in mice with periodontitis were associated with a decrease in SCFAs (e.g. butyrate) producing bacteria in the gut (Li et al., 2021). Also, butyrate supplementation reduced fasting glucose and insulin levels in mice and improved insulin sensitivity, the effects of which were mediated through inhibition of HDACs (McNabney and Henagan, 2017). Thus, reduced production of intestinal SCFAs may mediate the effect of periodontitis on diabetes. Furthermore, branched-chain amino acids (BCAA) are one of the links between periodontal bacteria and insulin resistance via the gut microbiota (Khor et al., 2021). Elevated levels of BCAA are characteristic of insulin resistance and have been suggested as a predictor of DM development (Pedersen et al., 2016; Nawaz and Siddiqui, 2020). Periodontal pathogenic bacteria (e.g. *P. gingivalis*) have been reported to alters the gut microbiota composition and serum metabolite profiles in mice, increasing BCAA levels and inducing insulin resistance (Tian et al., 2020). Mechanistically, BCAA activates the mTOR-S6K1 pathway, which induces insulin resistance by phosphorylating IRS-1 (Yoon, 2016).

Finally, gut dysregulation following swallowing of periodontal bacteria implies elevated levels of circulating LPS and induced/amplified systemic hypo-inflammation, which may be one of the potential mechanisms explaining the association between periodontitis and DM. Consistent with this, continuous infusion of *P. gingivalis*-LPS induced endotoxemia, glucose intolerance and insulin resistance in HFD-fed mice (Arimatsu et al., 2014; Blasco-Baque et al., 2017). Lowering circulating LPS concentration could be a potent strategy for the control of metabolic diseases. Gut microbiota dysbiosis may induce the expression of pro-inflammatory cytokines through the TLR4/MyD88/NF-κB signaling pathway, thereby promoting DM progression (Hou et al., 2022).

Gut dysbiosis and subsequent increased circulating levels of pro-inflammatory cytokines induced by swallowing periodontal bacteria may impair glycemic control in DM patients (Arimatsu et al., 2014; Hajishengallis and Chavakis, 2021). For example, chronic low-grade inflammation caused by periodontal bacteria can aggravate pancreatic β-cell dysfunction in DM mice through IL-12 regulation on Klotho, thereby worsening glucose control as well as glucose-stimulated insulin secretion (Liu et al., 2016). Studies have demonstrated that improved metabolic control (lower HbA1c and plasma glucose concentrations) after periodontal treatment is accompanied by a reduction in systemic inflammatory markers (D’Aiuto et al., 2018).

Rebuilding the gut microbiota may be an important way to inhibit the pathogenic effects of periodontitis on DM. Intervention studies using FMT in human and animal models have demonstrated the role of the gut microbiota in improving diabetes (Vrieze et al., 2012; Aron-Wisnewsky et al., 2019; Wang et al., 2020). Lean donor FMT in patients with metabolic syndrome showed altered gut microbiota and improved insulin sensitivity (Vrieze et al., 2012; Kootte et al., 2017). Gut microbiota from DM-resistant mice can also transfer DM protection to otherwise highly susceptible to DM hosts. A study reported that gut microbiota transfer from DM-protected MyD88-deficient non-obese diabetic (MyD88-/-NOD) mice could lead to delayed onset of DM and reduced insulitis in NOD mice (Peng et al., 2014). The study further found that the microbiota and mucosal immune system (elevated levels of IgA, TGF-β, etc.) were significantly altered in the gut of NOD mice after receiving the gut microbiota from DM-resistant mice (Peng et al., 2014). This suggests that inhibiting or reversing the alteration of the gut microbiota by periodontal bacteria might improve DM.

### 4.7 Atherosclerosis

AS is the pathological basis for the development of cardiovascular disease. Epidemiological studies have shown that periodontitis can significantly increase the risk and prevalence of atherosclerotic cardiovascular disease (ASCVD), including cardiovascular death, myocardial infarction, heart failure, atrial fibrillation and stroke (Linden et al., 2012; Dietrich et al., 2013; Chen et al., 2016; Liljestrand et al., 2017; Nordendahl et al., 2018; Park et al., 2019; Zhou et al., 2021). Periodontitis can also significantly increase the intima-media thickness (IMT) and atherosclerotic plaque area in the common carotid artery (Montenegro et al., 2019; Meurman and Söder, 2022). Many hypotheses have been proposed regarding the cause of AS, and it is now generally accepted that infection is an initiating factor and an important mechanism in promoting atherosclerotic changes in blood vessels (Slocum et al., 2016).

There are significant pro-inflammatory alterations in the gut microbiota of ASCVD patients, including reduced microbial diversity, altered F/B ratios and increased relative abundance of oral bacteria (Koren et al., 2011; Gregory et al., 2015; Emoto et al., 2016; Emoto et al., 2017; Jie et al., 2017; Yoshida et al., 2018). Relatively low abundance of SCFAs-producing bacteria and high abundance of LPS-producing bacteria have also been reported in the gut of ASCVD patients infected with periodontal bacteria (Arimatsu et al., 2014; Kramer et al., 2017). These intestinal alterations have not only been shown to predict and accelerate ASCVD, but have also been associated with dyslipidemia, elevated marker levels and a state of low systemic inflammation in ASCVD (Le Chatelier et al., 2013; Nie et al., 2019; Yeh et al., 2020). Conversely, FMT can reduce or transmit susceptibility to AS (Vrieze et al., 2012; Gregory et al., 2015). Quercetin has also been shown to modulate immune and metabolic function by restoring intestinal homeostasis, reducing the production of pro-inflammatory cytokines and atherosclerotic lipid metabolites, thereby significantly reducing areas of atherosclerotic lesions and sizes of plaques (Nie et al., 2019).

Pro-inflammatory alterations in the gut microbiota induced by periodontal bacteria may contribute to AS by modulating the production of its bioactive metabolites (Yeh et al., 2020). Plasma levels of trimethylamine N-oxide (TMAO) are an important predictor of adverse cardiovascular events, and a disordered gut microbiota can increase the conversion of TMAO (Tang et al., 2013; Wang et al., 2015). TMAO can inhibit reverse cholesterol transport to exacerbate foam cell accumulation, as well as induce platelet hyperactivity to increase thrombus formation, ultimately affecting atherosclerotic plaque formation and progression (Zhu et al., 2016; Yeh et al., 2020; Qian et al., 2022). In animal studies, transplantation of TMAO-rich gut microbiota feces into GF mice promotes platelet function and arterial thrombus formation (Huynh, 2020). In addition, TMAO also promotes the release of the inflammatory cytokines IL-18 and IL-1β by activating the NF-κB pathway (Liu M. et al., 2019).

The impact of infection on AS is now thought to be related to the total pathogen burden, i.e. the bacterial load determines the inflammatory status and stability of the atherosclerotic plaque (Emoto et al., 2016). Studies investigating the oral, gut and plaque microbiota in AS patients have confirmed that the atherosclerotic plaque microbiota is at least partly oral and gut in origin (Koren et al., 2011). The number and abundance of periodontal bacteria species detected in AS patients tends to be more significant and positively correlated with the size of atherosclerotic plaques (Ford et al., 2006; Otomo-Corgel et al., 2012; Rivera et al., 2013; Calandrini et al., 2014). Endotoxemia associated with the oral and gut dysbiosis may induce the recruitment of macrophages and their infiltration of adipose tissue, culminating in the formation of foam cells that accumulate in the artery walls (Diet, 2001; Michelsen et al., 2004; Mullick et al., 2005).

The pathogenic effect of periodontal bacteria on AS has been demonstrated in multiple types of animal model studies (Brodala et al., 2005; Velsko et al., 2014; Lin et al., 2015; Widziolek et al., 2016). A possible theoretical mechanism by which periodontal bacteria could reach the atherosclerotic plaque via the gut is currently mentioned, namely the phagocytosis by macrophages at epithelial linings of the gut (Schenkein and Loos, 2013; Emoto et al., 2016). Macrophage abundance and function can be regulated and driven by the gut microbiota and convey immune responses at distal organs (Yeh et al., 2020). periodontal bacteria are phagocytosed by macrophages in the dysregulated gut and carried into circulation. When they reach the activated endothelium of atherosclerotic plaques, they leave the bloodstream to enter the plaque and transform into cholesterol-laden foam cells (Wang et al., 2007; Emoto et al., 2016). *P. gingivalis* has been detected in circulating phagocytes from patients with severe cardiovascular disease suffering from periodontitis, supporting the possibility that phagocytes may play a role in harboring and transporting periodontal bacteria to atherosclerotic plaques (Carrion et al., 2012). However, the current study only demonstrates the theoretical validity of this periodontal bacteria transport mechanism and does not indicate the extent of its contribution in AS pathogenesis.

### 4.8 Skin diseases

Both the skin and the gut are important barrier structures for the immune and neuroendocrine functions of the body (O’Neill et al., 2016). It is now widely accepted that there is a close bidirectional relationship between the gut and the skin, and a “gut-skin axis” has been proposed to further explore the link between gut and skin homeostasis (Salem et al., 2018).

A number of studies have referred to preliminary evidence that the oral and gut microbiota may play a role in the pathogenesis of skin diseases as regulators of the “gut-skin axis”. Sjögren’s syndrome, psoriasis, systemic lupus erythematosus (SLE) and leukoaraiosis may all be linked to dysbiosis of the oral and gut microbiota (Szymula et al., 2014; Consolandi et al., 2015; Coit et al., 2016; Ungprasert et al., 2017; Ma and Morel, 2022). Some oral microbiota species are enriched in the gut of SLE patients. In both SLE and psoriasis, the severity of skin lesions, including the SLE Disease Activity Index and the Psoriasis Area and Severity Index, were positively correlated with the severity of periodontitis and altered gut microbiota profiles (Masallat and Moemen, 2016; Azzouz et al., 2019; Qiao et al., 2019; Hussain et al., 2022).

In addition, elevated levels of markers of intestinal barrier function impairment are often observed in a variety of skin diseases. elevated levels of fecal calprotectin have been found in patients with SLE and atopic dermatitis (Seo et al., 2018; Azzouz et al., 2019; Smirnova et al., 2020). Elevated levels of claudin-3 and intestinal fatty acid-binding protein have also been found in the blood of patients with psoriasis (Sikora et al., 2019a; Sikora et al., 2019b). A compromised gut barrier allows periodontal or other bacteria to move from the gut lumen into the internal environment where they can accumulate in the skin and disrupt skin homeostasis. Studies have detected periodontal bacteria and gut-derived bacterial DNA in the blood of patients with psoriasis, while finding that those with bacterial DNA-positive psoriasis patients showed higher serum inflammatory cytokine levels, longer duration of disease and younger onset of disease (Ramírez-Boscá et al., 2015; Dalmády et al., 2020). In Sjögren’s syndrome, Sjögren’s syndrome Antigen A (SSA)/Ro60-reactive T cells can be activated by peptides originating from oral and gut bacteria, and thus may be involved in disease progression (Szymula et al., 2014).

Periodontal bacteria-induced gut dysbiosis may be involved in the pathogenesis of autoimmune (e.g. psoriasis) and allergic (e.g. atopic dermatitis) skin diseases through immune, metabolic and neuroendocrine pathways. First, disruption of Th17/Treg balance can be involved in the pathogenesis of several chronic inflammatory skin diseases (Salem et al., 2018). In particular, psoriasis is mainly considered to be a Th17 disease (Grine et al., 2015). Activation of Th17 cells and increased expression of IL17 are major factors mediating the pathogenesis of psoriasis, which could be induced by periodontal bacteria and their products (Blauvelt and Chiricozzi, 2018; Bunte and Beikler, 2019; Zhao X. et al., 2021). After antibiotic treatment, reduced skin inflammation caused by reduced Th17 activation in the gut was observed in a mouse model of imiquimod-induced psoriasis (Zákostelská et al., 2016). It was further shown that IL-17 disrupts the integrity of the skin barrier through downregulation of filaggrin and adhesion molecule expression from keratinocytes, and further induces keratinocyte hyperproliferation (Gutowska-Owsiak et al., 2012). Thus, systemic Th17 hyperactivation or overproduction of IL-17 may be the link between periodontal bacteria-induced disturbances in the gut microbiota and the development of psoriasis. In addition to this, skin diseases such as Sjögren’s syndrome, SLE, leukoplakia and scleroderma have also been linked to the pathogenic mechanisms by which periodontitis regulates Th17 cell differentiation and its key cytokines (IL-17 and IL-23) (Bunte and Beikler, 2019; Tong et al., 2019).

Secondly, periodontal bacteria may mediate skin inflammation by affecting the normal metabolic activity of the intestinal commensals. For example, elevated serum levels of free phenol and p-cresol may affect epidermal differentiation and epidermal barrier function by decreasing keratin 10 expression (Miyazaki et al., 2014). Both oral and gut microbiota dysbiosis in SLE patients may be related to amino acid metabolism. In SLE progression, amino acid (e.g. tryptophan) metabolism can modulate immune tolerance in lupus. We can therefore speculate that alterations in the oral and gut microbiota of SLE patients with periodontitis may initiate autoimmunity by manipulating amino acid metabolism. However, this needs to be verified by further studies.

Finally, some studies support the idea that alterations in the gut microbiota can be involved in the development of skin diseases by affecting the levels of circulating neuroendocrine molecules such as tryptamine, trimethylamine and serotonin, inducing skin barrier dysfunction and immune system dysregulation (Jin et al., 2014; O’Neill et al., 2016).

## 5 Discussion

This review suggests that periodontal bacteria gain access to systemic dissemination through the disturbed gut, thereby modulating the host’s inflammatory state and immune function, and ultimately the development of multiple systemic diseases (**Table 1**). The downside is that this article may not have listed all the systemic diseases that may be associated with the pathogenic mechanisms of the oral-gut axis in the setting of periodontitis, such as lung disease and other cancers of the digestive tract such as pancreatic cancer. Some diseases, such as adverse pregnancy outcomes, have been shown to be strongly associated with both periodontitis and the gut microbiota, but no studies have yet reported whether their development may be influenced by the intestinal transit of periodontal bacteria. We hope that in the future more and more evidence will emerge to help us identify more systemic diseases that may have oral-gut transit of periodontal bacteria involved in the pathogenic mechanism. In addition, this paper uses a wealth of evidence to interpret the causal role of periodontal bacteria in a variety of systemic diseases through the oral-gut axis, the oral-gut-liver axis and the oral-gut-brain axis in the pathogenesis of disease. This suggests a potential therapeutic and preventive route by blocking the transmission route. However, periodontal bacteria may also play a pathogenic role in distal organs through other parenteral routes. Further studies are needed to elucidate whether indirect regulation of periodontal bacteria through the oral-gut route is predominant.

**Table 1:** Impact of periodontal bacteria on systemic diseases via the oral-gut axis.

Systemic Diseases	Periodontal Bacteria	Possible Pathogenic Mechanisms Through the Oral-gut Axis	Impact on Systemic Diseases
Inflammatory Bowel Disease (IBD)	*P. gingivalis, F. nucleatum, P. intermedia*	(i) compromise the intestinal barrier (downregulation of TJs), enhance intestinal immune responses (e.g. induction of M2 macrophage polarization), and affect intestinal metabolism (e.g. decreased unsaturated fatty acid synthesis and increased arachidonic acid metabolism)	Promote the development of IBD and exacerbate the progression of pre-existing IBD
		(ii) Induction and migration of oral pathobiont-reactive Th17 cells indirectly exacerbate intestinal inflammation.	
Colorectal Cancer (CRC)	*P. gingivalis, F. nucleatum*	exploit tumor surface barrier defects, invade normal colonic tissue and induce local inflammation, while producing genotoxic metabolites to induce oncogenic transformation of colonic epithelial cells.	Promote tumor progression, metastasis and recurrence
Rheumatoid Arthritis (RA)	*P. gingivalis, P. intermedia, A. actinomycetemcomitans, F. nucleatum, P. nigrescens*	(i) Increased production of pro-inflammatory factors (e.g. IL-6) and citrullinated protein in joint and intestinal tissues disrupts immune tolerance in susceptible individuals.	Exacerbate the severity of joint destruction and affect regression on anti-rheumatic drugs
		(ii) trigger synovial inflammation by activating the FadA-Rab5a-YB-1 axis in synovial macrophages.	
		(iii) inflammatory bone destruction by increasing the intestinal hyperimmune response triggered by Th17 cellular immune responses.	
Alzheimer’s Disease (AD)	*P. gingivalis, T. denticola*	Promote neuroinflammation and neurodegeneration by disrupting the intestinal barrier and BBB. This includes inducing Aβ plaque formation through activation of the TLR4/NF-κB and MAPK pathways, proteolysis of tau and subsequent NFTs formation through activation of caspase-3, and neuronal death through triggering of the immune cascade response.	Increase risk of developing AD and increased dementia severity (e.g. cognitive impairment)
Parkinson’s Disease (PD)	*P. gingivalis*	(i) Disrupt the intestinal barrier and BBB and activate the TLR4/MyD88/NF-κB pathway in the SN and colon, ultimately leading to dopaminergic neuronal damage and microglia activation in the SNpc.	Increase risk of developing PD and correlate with disease severity
		(ii) Induce misfolding and aggregation of α-synuclein in specific enteric neurons and subsequent migration to the brain.	
Autism Spectrum Disorder (ASD)	*P. gingivalis*	(i) Disrupt the intestinal barrier and BBB, triggering the microglia-associated NF-κB pathway to induce neuroinflammation.	Correlate with ASD severity
		(ii) Promote mitochondrial dysfunction, resulting in alterations in metabolism and neurosignaling activities.	
Major Depressive Disorder (MDD)	*P. gingivalis, F. nucleatum, A. actinomycetemcomitans*	(i) Disrupt the intestinal barrier and the BBB, leading to high activation of astrocytes and promoting neuroinflammation and neuronal death.	increase risk of developing MDD, induce depression-like behavior and correlate with disease severity
		(ii) potential association on the transcriptomic level.	
Liver Disease	*P. gingivalis, A. actinomycetemcomitans, T. denticola*	activate the PRR via the oral-gut-liver axis (e.g. portal circulation) and act directly on hepatocytes, Kupffer cells and HSC, triggering downstream pro-inflammatory cascades and ultimately inflammation and fibrosis in the liver.	affect the prevalence, mortality, disease severity and prognosis of many chronic liver diseases (e.g. NAFLD, NASH and cirrhosis)
Obesity	*P. gingivalis, A. actinomycetemcomitans, T. forsythia*	(i) induce insulin resistance, hepatic steatosis and macrophage infiltration in adipose tissue.	Increase the risk of obesity, correlate with disease severity, and affect regulation of energy use and metabolism *in vivo*
		(ii) Induced endotoxemia may alter endocrine function and gene expression in BAT.	
		(iii) affect the secretion of pro/anti-inflammatory adipokines.	
Diabetes Mellitus (DM)	*P. gingivalis, A. actinomycetemcomitans, T. forsythia, F. alocis*	induce/exacerbate insulin resistance and glucose intolerance by mediating entero-hepatic metabolic derangements.	Increase the risk of developing DM and affect glycemic control in DM patients
Atherosclerotic Cardiovascular Disease (ASCVD)	*P. gingivalis, A. actinomycetemcomitans, T. denticola, T. forsythia, F. nucleatum, P. intermedia*	(i) modulate the production of bioactive metabolites (e.g. TMAO) from the gut microbiota and influence atherosclerotic plaque formation and progression.	Increase the risk of ASCVD, promote inflammatory and atherosclerotic changes in blood vessels.
		(ii) induce the recruitment of macrophages and their infiltration of adipose tissue, culminating in the formation of foam cells that accumulate in the artery walls.	
Skin disease	*P. gingivalis, A. actinomycetemcomitans, P. intermedia, T. denticola, T. forsythia, P. nigrescens*	modulate autoimmune and allergic skin diseases through immune (e.g. Th17/Treg balance), metabolic, and neuroendocrine pathways.	correlate with severity of skin disease

Dysbiosis of the oral microbiota is closely associated with the development and progression of many systemic diseases. In recent years there has been an increasing interest in therapeutic interventions to regulate and re-establish the homeostasis of the human oral microbiota and thereby restore systemic health. Inspired by FMT, oral microbiota transplantation (OMT) has started to be considered by researchers as a promising treatment for improving oral and extra-oral diseases. The OMT procedure recommended in the current study consists of (i) collection of supra/subgingival plaque from a healthy donor followed by *in vitro* culture to obtain a sample with the healthiest oral microbiota, (ii) pre-treatment of the recipient’s oral cavity, e.g., physical plaque removal and the application of antimicrobial agents, and lastly (iii) transplantation of the donor oral microbiota is accomplished by rinsing/applying (hydrogel may be considered as a delivery vehicle) (Beikler et al., 2021; Nath et al., 2021; Xiao et al., 2021; Weyrich et al., 2024). The oral microbiota appears to be more stable than other body niches (e.g. the gut), but at the same time oral biofilms display significant phenotypic plasticity (microbial community composition fluctuates in response to changes in the oral environment). Introducing health-associated oral microbiota into the oral cavity of a diseased patient is a key factor in the potential therapeutic role of OMT by shaping and altering the composition and function of the human microbial community, which in turn affects systemic health and disease states.

The potential of OMT in the treatment of oral diseases (e.g. dental caries and periodontitis) has now been documented (Pozhitkov et al., 2015; Nascimento, 2017; Nath et al., 2021). Beikler et al. validated the safety and efficacy of OMT in the treatment of periodontitis in dogs (Beikler et al., 2021). The findings suggest that a single OMT from a healthy periodontal donor as an adjunct to mechanical and chemical full-mouth debridement has a greater modulating effect on the oral microbiota composition in dogs with naturally occurring periodontitis than full-mouth mechanical and antimicrobial debridement alone. In addition, using an experimental periodontitis mouse model, Xiao et al. showed that OMT from healthy to irradiated mice reduced OM-related epithelial damage and oral and systemic inflammation by attenuating irradiation-induced alterations in oral and gut microbiota (Xiao et al., 2021). The results of this study support the mechanism of the oral-gut axis and confirm that OMT may be employed as a novel therapeutic avenue for radiation-induced oral mucositis of head and neck cancer patients after radiotherapy in preclinical settings (Xiao et al., 2021). However, more *in vivo* studies are still needed to confirm the safety and efficacy of OMT in the treatment of human disease. In the meantime, OMT is only an adjunct to the treatment of systemic disease and patients will need to take other therapeutic measures to manage and treat the disease.

This article illustrates the far-reaching implications of maintaining periodontal health in reducing the risk of many intestinal and extraintestinal diseases. Some studies have already been conducted on periodontal treatment or modification of the oral microbiota as an entry point for the treatment and prevention of systemic diseases, such as targeted inhibitors against virulence factors of periodontal bacteria to reduce the ability of bacteria to translocate to distal tissues. However, more research is still needed in the future to investigate the role of periodontitis in the regulation of systemic disease pathogenesis through the oral-gut axis and to clarify the importance of oral microbiota defense in building systemic microbiota immunity. This may provide insight into the underlying pathogenesis of many systemic diseases and the search for new preventive and therapeutic strategies.
